# Mindfulness-based (non-contact) boxing therapy (MBBT) for depression and anxiety: A feasibility study

**DOI:** 10.1371/journal.pone.0318364

**Published:** 2025-02-06

**Authors:** Johny Bozdarov, Brett D.M. Jones, Madeha Umer, Daniel M. Blumberger, Ishrat M. Husain

**Affiliations:** 1 Department of Psychiatry, Temerty Faculty of Medicine, University of Toronto, Toronto, Ontario, Canada; 2 Campbell Family Mental Health Research Institute, Centre for Addiction and Mental Health (CAMH), Toronto, Ontario, Canada; 3 Institute of Medical Sciences, Temerty Faculty of Medicine, University of Toronto, Toronto, Ontario, Canada; 4 Temerty Centre for Therapeutic Brain Intervention, CAMH, Toronto, Ontario, Canada; Universidad de Valencia and University of Bologna, SPAIN

## Abstract

**Objectives:**

Mindfulness-Based (non-contact) Boxing Therapy (MBBT) is a novel intervention designed to empower and promote self-agency through behavioral interventions, while reducing barriers to exercise for individuals with mental disorders. MBBT is an instructor-led, manualized, non-contact boxing group-exercise program (delivered in 90 min sessions, twice a week, over 10 weeks) that blends principles of mindfulness, meditation and group therapy. The current study tested the acceptability and feasibility of delivering MBBT to adults with major depressive disorder (MDD) or generalized anxiety disorder (GAD).

**Methods:**

Nine adult outpatients with MDD or GAD were recruited from a psychiatric outpatient clinic in Toronto, Canada in a 10-week feasibility trial of MBBT using a pre-post design. Feasibility was assessed through recruitment and retention rates, while acceptability was assessed through the CSQ-8, and self-questionnaires. Secondary clinical outcomes included the PHQ-9, GAD-7, K10, CGI, and MAAS. Trial registry: ISRCTN23023309.

**Results:**

Eight participants (5 female, 3 male) were included in the final analysis. Results indicated a high user retention (89%), attendance (84%), and satisfaction (98%). The study observed a statistically significant mean percent reduction in depression (54%), anxiety (51%) and distress (36%), alongside a mean percent increase in mindfulness (79%). Post intervention qualitative feedback from participants revealed themes of inclusivity and accessibility, cathartic release and control of emotions, improved self-esteem and confidence, self-agency, community, and trust in leadership.

**Conclusions:**

Given the limitation of the study, MBBT appeared to be feasible and acceptable as an exercise/behavioural intervention. Further well-designed randomized clinical trials are warranted to confirm the clinical benefits of MBBT.

## Introduction

Mental disorders are a leading cause of the global burden of disease, with depression and anxiety being the most prevalent among them with substantial individual and societal costs [1,2]. With a growing awareness of mental well-being and increasing rates of psychological distress, depression, and anxiety worldwide, it is evident that there is a need for more accessible and effective support for mental health [2,3]. The optimal approach to treating mental disorders encompasses a bio-psycho-social model, recognizing that biological, psychological, and social factors contribute to the development of most conditions [4,5]. Medication and psychotherapy remain the primary focus of treatment with pharmacotherapy often being the predominant treatment approach for depression and anxiety in practice [4,5]. Lifestyle interventions (e.g., exercise, behavioral interventions, and mindfulness meditation) are often labeled as as “complementary” or “alternative” yet have good evidence and are particularly relevant as many patients prefer to avoid the significant side effect burden associated with psychotropic medication [6,7]. Limited supervised support programs and associated costs are the most prominent barriers that reduce exercise adherence amongst patients [8,9]. The importance of these interventions is underscored by the increased risk of premature mortality from metabolic and cardiovascular complications — including diabetes, cardiovascular disease, and obesity — that disproportionately affects individuals with mental health conditions such as depression [10–13].

There is replicated evidence for the mental health benefits of all modes of physical activity to address mild-to-moderate symptoms of major depressive disorder (MDD), anxiety, and psychological distress, alongside improvements in quality of life in these populations [14–17]. For example, aerobic exercise incorporating high-intensity-interval training (HIIT) is superior to low intensity training in improving depressive symptoms [14,18,19]. In addition, exercise interventions (i.e., a planned, structured, repetitive and purposive physical activity) appear to be non-inferior to current first line treatments for depression with group aerobic exercises of moderate intensity being the most efficacious [20]. Physical activities that incorporate psychotherapeutic techniques such as mindfulness report better outcomes for mental health than those that are not [21], specifically around anxiety [14]. This finding is not particularly surprising as mindfulness meditation interventions (e.g., Mindfulness-Based Stress Reduction (MBSR)) have shown to be an effective treatment modality for depression and to be equivalent to first line pharmacotherapy for the treatment of generalized anxiety disorder (GAD) [22,23]. Taken together, evidence suggests that physical activity, in particular those that incorporate psychotherapeutic techniques such a mindfulness, and behavioral interventions, can play a role in reducing the disability associated with mental disorders.

Non-contact boxing is a form of exercise that emphasizes the physical fitness and skill-building aspects of boxing without actual physical contact or sparring with opponents. Non-contact boxing often occurs in a supervised, group setting, utilizing a HIIT circuit involving shadowboxing and striking a heavy bag or hand pads. Non-contact boxing often uniquely encompasses both aerobic (i.e., HIIT training) as well as body awareness, and deep breathing during recovery. Striking a heavy bag or hand pads also provides a uniquely cathartic release of emotions such as anger, aggression, and stress [24]. A recent scoping review reported numerous mental health benefits of non-contact boxing including improved mood, reduced anxiety, increased self-esteem, self-agency, confidence, concentration, and strength [24]. Despite these potential benefits, there is a gap in the integration of non-contact boxing exercises with mindfulness interventions in a structured therapeutic program for individuals with mental health problems.

Mindfulness-Based (non-contact) Boxing Therapy (MBBT) is a novel intervention designed to empower and promote self-agency through behavioral approaches. MBBT is an instructor-led, manualized, non-contact boxing group-exercise program (delivered in 90 min sessions, twice a week, over 10 weeks) that blends principles of mindfulness, meditation and group therapy. Participants learn the foundations of boxing and mindfulness principles and advance them each week instilling self-agency and growth towards independent practice when the therapy finishes. MBBT aims to reduce barriers to instructor-led exercise programs while decreasing the disability associated with mental disorders, such as depression and anxiety.

The present study aimed to assess the feasibility and acceptability of MBBT as a novel intervention for patients with MDD and GAD. This is an important first-step before implementing a randomized controlled trial (RCT) to assess recruitment, retention, and outcomes of the intervention [25,26].

## Methods

### Study design

A 10-week, single arm, feasibility trial of MBBT using a pre-post design utilizing a mixed-method approach by including both measurement based-scales for quantitative data as well as qualitative analysis relating to expectations and feedback. The study was conducted in person at the Centre for Addiction and Mental Health (CAMH), a large academic psychiatric hospital in Toronto, Canada.

The CAMH Research Ethics Board approved the study protocol, all supplementary documentation, and the informed consent form (REB #153/2021). Prior to the initiation of any study procedures, participants received both verbal and written information about the study and provided written informed consent. Informed consent, intake assessments, and orientation were all conducted virtually using the secure WebEx platform. Participants had the option to withdraw from the study at any time upon their request, at the request of the treatment team, or if they experienced a significant worsening of symptoms. Participant records were securely stored.

Symptoms measurement was completed at assessment, midway, and post-intervention when applicable. Upon completion of the study, participants were able to keep the personal equipment used (i.e., boxing gloves, hand wraps, skipping rope, water bottle, yoga block and yoga mat) and were compensated for transportation costs.

**Fig 1:**
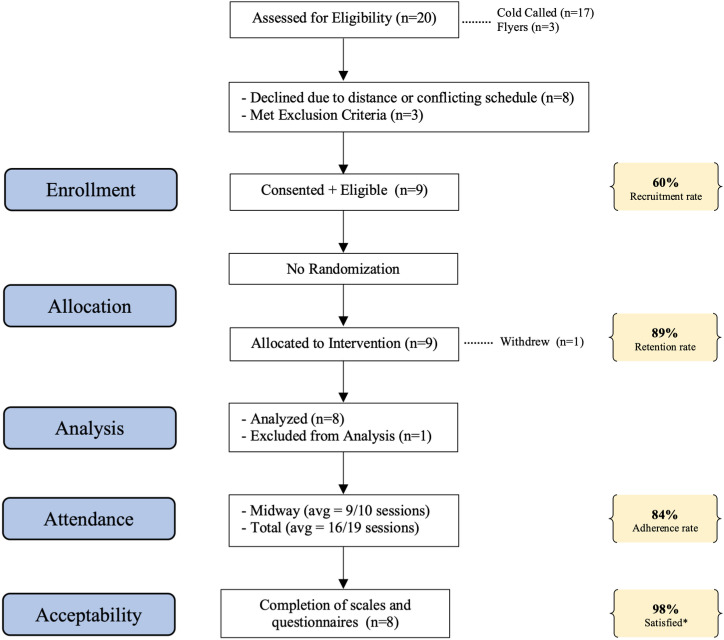
CONSORT flow-chart [Flow of participant inclusion and feasibility/acceptability measures]. The asterisk (*) indicates satisfaction as measured by the CSQ-8.

Due to the time-sensitive and uncertainties presented by the COVID-19 pandemic, administrative challenges required us to proceed with participant recruitment swiftly, resulting in the trial being retrospectively registered (ISRCTN23023309). The protocol for this trial, ethics approval and TREND checklist are available as supporting information. Fig 1 illustrates the flow and progression of participants through the study.

### Mindfulness-based boxing therapy

MBBT was delivered by a trained instructor (JB) at a hospital gymnasium over a 10 week period, twice a week. The intervention ran continuously from November 8, 2022 to January 13, 2023. E-mail reminders were sent to participants prior to each session.

**Fig 2:**
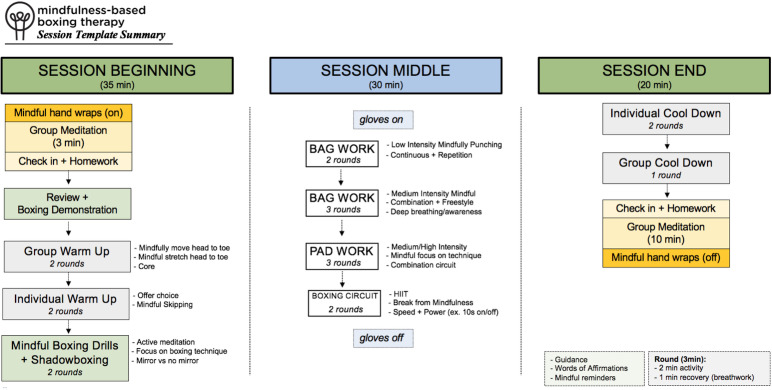
Brief breakdown of an average MBBT Session.

The supporting material (S1 File) provides an outline of the overall breakdown of the beginning, middle, and end phases of MBBT alongside core principles. A breakdown of the timeline of a typical session of MBBT is outlined in Fig 2.

Each session starts and ends with meditation in the form of mindful hand wrap activity and a sitting meditation. Followed by checking in on homework and delivering psychoeducation plus the mindfulness boxing activity for the day. The session would then move towards individual and group warm-up with a focus on mindful aerobic cardio, such as skipping or shadowboxing, and light stretching using yoga poses. Once the boxing gloves were on, the focus was on a build-up of light to high-intensity circuits incorporating boxing bags, and hand pads. The cool down period mimics the warmup period with light cardio, shadowboxing, and stretches both individually and in a group.

Music was played at each phase of the intervention beginning with meditation music, transitioning gradually to higher-intensity music, and concluding with meditation music. There was flexibility in terms of exercise choice to enhance personalization and accessibility.

### Sample size, study population, and recruitment

A sample of 9 participants were recruited due to institutional COVID-19 restrictions of a maximum of 10 person gatherings. Usually a size of 12 is recommended for a feasibility study to obtain appropriate data [27] but infection control limitation prevented this and facilitating a second MBBT cohort was not possible due to limited resources.

Eligible participants were aged 18 to 40 years with a current primary diagnosis of either MDD or GAD determined by a structured psychiatric diagnostic interview performed by a trained clinician. All participants were enrolled from the Mood Disorders Services (MDS) at CAMH who had previously consented to be contacted for research purposes. Recruitment occurred by phone and flyers placed within CAMH outpatient services. Recruitment occurred from September 29, 2022 to November 6, 2022.

Eligible participants were: (1) outpatients; (2) competent to consent to treatment; (3) a DSM–5 diagnosis of non-psychotic MDD, single or recurrent, or GAD; (4) male or female between the ages of 18 - 40; (5) able to adhere to the study schedule; (6) taking psychiatric medication for at least 4 months – but were not planning on initiate a new treatment regimen during the study.

Participants were excluded if they: (1) met DSM-5 substance use disorder criteria within the past three months; (2) had a concomitant major unstable medical illness; (3) were pregnant or intend to get pregnant during the study (confirmed verbally); (4) had a SCID-5 diagnosis of any psychotic disorder, bipolar disorder, obsessive compulsive disorder, or post-traumatic stress disorder (current or within the last year); (5) had a DSM-5 diagnosis of borderline personality disorder; (6) had prior history of violence or sexual aggression; (7) planned on enrolling in another psychosocial intervention or psychotherapy during the study - but could continue with one if they were engaged with it prior to recruitment.

### Study objectives, measures and outcomes

The primary aim was to determine whether MBBT added to standard care was feasible and acceptable for patients with MDD and/or GAD. Feasibility measures included recruitment, retention, and adherence. MBBT acceptability was quantitatively measured using the Client Satisfaction Questionnaire (CSQ-8), a self-reported scale consisting of 8 items that assess global satisfaction with a service [28] and a User-Experience Questionnaire.

The secondary aim was to determine whether MBBT led to a reduction of mental health symptom burden. The 9-item Patient Health Questionnaire (PHQ-9) [29] was used to assess depressive symptoms, the Generalized Anxiety Disorder-7 (GAD-7) [30] scale was used to assess anxiety, the Kessler Psychological Distress Scale (K10) [31] was used to measure psychological distress and the Clinical Global Impression of Severity (CGI-S) [32] was used to assess overall severity of symptoms.

The exploratory aim was to measure the level of mindfulness experienced by participants of MBBT using The Mindful Attention and Awareness Scale (MAAS) [33] and State Mindfulness Scale for Physical Activity (SMS-PA) [33]. Given that this is an exercise intervention, we were also interested in exploring any changes in Body Mass Index (BMI) and qualitative changes related to fitness.

### Qualitative data analysis

An inductive analysis was conducted from the transcribed records within session, voluntary e-mail feedback, and from the User-Experience Questionnaire. A thematic approach as per Braun and Clarke’s 15-point checklist of criteria for good thematic analysis [34] was applied. The transcribed records were coded and themes were identified independently by a member of the study team (MU). Author JB developed a consensus summary of qualitative themes. The final qualitative analysis was reviewed by a senior author (MIH).

### Quantitative data analysis

Statistical analysis was completed using Microsoft Excel Software and R statistical software on R Studio for Windows 10. The mean, percentage changes, and Wilcoxon’s Signed-Ranked test between the pre and post means were calculated alongside Cohen’s d effect size. Descriptive statistics were used to present demographic data, recruitment, retention, acceptability and other outcomes.

## Results

### Baseline characteristics

Table 1 summarizes the demographic and clinical characteristics of enrolled participants.

**Table 1:** Participant demographics and clinical characteristics.

CHARACTERISTICS	MBBT PARTICIPANTS (n = 8)
**Demographics**	
Age - mean years (SD)	28.9 (5.6)
Primary Language -% English (n)	100% (8)
Sex - % female (n)	65% (5)
Race - % Caucasian (n)	87% (7)
Education - % post-secondary (n)	75% (6)
Employment - % full time (n)	50% (4)
Relationship - % single (n)	50% (4)
Dependents - % with (n)	13% (1)
Pharmacotherapy - % taking (n)	75% (6)
**Psychiatric Diagnosis**	
% MDD (n)	100% (8)
% GAD (n)	87% (7)
% SAD (n)	13% (1)
% Trauma History (n)	50% (4)

### Safety considerations

There were no serious adverse events. Minor adverse events included some wrist strain and abrasions from boxing.

### Feasibility and acceptability measures

Fig 1 summarizes the feasibility and acceptability measures. Of the 20 patients approached, 12 completed informed consent (60% consent rate). Reasons for declining participation included distance (50%) or conflicting work/school schedule (50%). Nine participants met criteria for eligibility (6 female and 3 male). Reasons for exclusion were related to concurrent diagnosis.

Of the 9 participants enrolled, one withdrew during week 6 without providing a reason, resulting in a retention rate of 8/9 (89%). Only data from the 8 participants completing the trial were included in the results. All 8 participants completed all scales and questionnaires.

The mean attendance at the midway point of the trial was 9/10 (90%) with a mean attendance of 16/19 (84%) by the end of the trial. One session was canceled due to inclement weather.

All participants completed the CSQ-8 questionnaire (possible range 8-32, higher scores indicate higher satisfaction) with a mean of 31.5/32 indicating a 98% overall high satisfaction with the intervention. All participants completed the User Experience Questionnaire and indicated extreme satisfaction with the intervention with all participants (100%) indicating they have plans to continue practicing MBBT on their own.

### Clinical outcomes

Table 2 summarizes the clinical outcomes and statistical analysis. The mean baseline PHQ-9 score was 17.5 (SD 3), indicating moderate to severe depression, and the post-treatment score was 8.1 (SD 3), indicating mild depression, with 54% reduction in overall depressive symptoms (p =  0.014). With a response rate of 50% and a remission rate of 13%. The mean baseline GAD-7 score was 12.5 (1.7), indicating moderate anxiety, and the post-treatment score was 6.1 (SD 2.1), indicating mild anxiety, with a 51% reduction in overall anxiety symptoms (p =  0.014). With a response and remission rate of 38%. Response was defined as a 50% or greater reduction in scores, while remission was indicated by scores below 5, for both PHQ-9 and GAD-7 assessments. Baseline K10 score was 34.1 (3.7), indicating very high psychological distress and likelihood of having a severe mental disorder, and post-treatment score was 21.9 (5.2), indicating high psychological distress and likelihood of having a mild mental disorder, with a 36% overall reduction in distress (p =  0.014). With regards to mindfulness, the change in MAAS scores pre-to-post treatment indicated a 79% overall mean increase in mindfulness (p =  0.007). As per SMS-PA, the overall state mindfulness during session 5 was 73% and during session 19 was 80%. There was an overall global clinical improvement of 34% as per CGI (p =  0.013). There was a 0.02% decrease in BMI which was not significant (p =  0.09).

**Table 2:** Summary of clinical measures.

CLINICAL MEASURES	MBBT PARTICIPANTS (n = 8)
**PHQ9**	
Prestudy mean (SD)	17.5 (3)
Midway mean (SD)	12 (2.6)
Poststudy mean (SD)	8.1 (3.7)
End change mean (SD)	**−9.4 (2.1)** *
End Percent change (%)	**−54%**
P-value	0.014
Cohen’s d effect size	2.79
**GAD7**	
Prestudy mean (SD)	12.5 (1.7)
Midway mean (SD)	8.6 (2.7)
Poststudy mean (SD)	6.1 (2.2)
End change mean (SD)	**−6.4 (2.2)** *
End Percent change (%)	**−51%**
P-value	0.014
Cohen’s d effect size	3.26
**K10**	
Prestudy mean (SD)	34.1 (3.7)
Midway mean (SD)	26 (5.2)
Poststudy mean (SD)	21.9 (5.2)
End change mean (SD)	**−12.3 (4.9)** *
End Percent change (%)	**−36%**
P-value	0.014
Cohen’s d effect size	2.7
**CGI**	
Prestudy mean (SD)	3.62 (0.7)
Midway mean (SD)	2.5 (1.1)
Poststudy mean (SD)	1.25 (0.5)
End change mean (SD)	**−2.4 (0.7)**
End Percent change (%)	**−34%** *
P-value	0.013
Cohen’s d effect size	3.9
**MAAS**	
Prestudy mean (SD)	2.3 (0.5)
Midway mean (SD)	3.1 (0.7)
Poststudy mean (SD)	3.9 (0.4)
End change mean (SD)	**1.6 (0.6)** *
End Percent change (%)	+**79%**
P-value	0.007
Cohen’s d effect size	3.53
**BMI**	
Prestudy mean (SD)	29 (14.2)
Midway mean (SD)	28.5 (13.9)
Poststudy mean (SD)	28.6 (13.7)
End change mean (SD)	**−0.4 (0.7)**
End Percent change (%)	**−2%**
P-value	0.09
Cohen’s d effect size	0.03

*p = <0.05 (Wilcoxon’s signed-ranked test between pre-post).

### Qualitative results

Acceptability themes from the qualitative analysis revealed themes of inclusivity and accessibility, cathartic release and control of emotions, improved self-esteem and confidence, self-agency, community and trust in leadership (Textbox 1).

The responses from participants regarding accessibility issues consistently convey a shared sentiment of minimal to no accessibility challenges. Most participants simply responded with a succinct “No” or “Not at all,” indicating that they did not encounter any barriers or difficulties in accessing the intervention, or with the structure or delivery time. They all felt it was feasible. All participants commented that they felt it was a safe and inclusive environment. Some barriers that were discussed including the inclement weather, transportation, and taking time away from a dependent.

MBBT was largely regarded as beneficial as an outlet for stress or anger by the participants with an overall increase control of emotions. Some participants stated that the program provided them with a healthy outlet for stress management, and one participant felt it allowed them to avoid self-harm or destructive behaviors. They noticed progress in their mindfulness practices, which assisted them in grounding themselves during stressful situations and becoming more aware of their emotions.

Participants’ emphasized a strong sense of community within the group setting. This atmosphere allowed participants to express their emotions, channel their aggressiveness in a safe and constructive way, and build a sense of belonging. All participants commented on the group dynamic and instructor as a large motivating factor that influenced cohesiveness.

In addition, participants discussed a strong desire to continue practice using words like “highly likely” and “very likely.” The enjoyment of the intervention was recognized as a motivating factor by these participants, indicating a pleasant experience that encourages them to keep involved. The last week of MBBT encourages and guides participants towards independent practice.

Textbox 1 **Qualitative themes and representative quotes**

**Theme 1: Inclusivity and accessibility**

“everyone being non-judgemental and being themselves. I felt safe and like we were family”
“helped me get out of my funk [...] having a safe space to workout”
“felt very safe”
“you gave different alternatives that were better for me and my body”

**Theme 2: Cathartic Release and Control of Emotions**

“it helped me use activity and boxing instead of more self-harm/destructive tendencies. I noticed when I missed a class these things relapsed”
“gave me an outlet twice a week, was great for stress”
“decreased stress and anger”
“it’s been really life changing to have different coping mechanism”
“I can recognize my stress and deal with it, and being kind to myself.”
“it has been incredibly helpful in giving me tools to identify when I am not in the moment and dealing with strong judging emotions”

**Theme 3: Improved Self-Esteem and Confidence**

“I have very rarely in the last 11 years showed up for myself [...] the people I love have seen a change in me [...] I missed smiling, laughing. I missed me and I feel like I’m slowly but surely getting back!”
“I’ve noticed an improvement in both my self-esteem and overall confidence.”
“it increased my confidence and has been a key component in my recovery. I felt proud I was able to attend and put my all into something”
“so much more confident and social”
“given me a quiet confidence in all aspects of life”

**Theme 4: Self-Agency**

“getting through tough workouts, and seeing payoff in the form of positive physical and mental results gave me confidence, and knowing that I’m a physically more capable human feels great for my self-esteem”
“I have a new lease on thinking and tools to encourage change”
“it showed me though I’m in crisis, I can still achieve things”
“helped me realize the power of breathing through stress and enjoying moments”

**Theme 5: Community + Leadership**

“group dynamic is very important, feeling like I was never judged. Very supportive group”
“great group of open individuals that made it feel safe”
“most important part was Johny, he provided a safe inclusive space”
“Johny’s ability to make such a positive environment was by far the safest I’ve ever felt in a fitness environment”

## Discussion

This is the first presentation of a novel group and mindfulness-based exercise intervention. We found that MBBT is a feasible and acceptable intervention for adult outpatients experiencing symptoms of anxiety and depression as demonstrated by the strong recruitment and retention rates and qualitative feedback from participants. The strong recruitment rate of 60% within a 5-week period indicates interest in and willingness to participate in such a study. Recruitment rates vary significantly in clinical trial research with suggestions of rates above 50% indicating a high level [35]. The high recruitment rate may be due to factors that in general aid in recruitment, such as multiple platforms, the novelty of the intervention, the lack of control group, the free access to non-contact boxing training, and the financial incentive [35–37].

Despite challenges, such as inclement weather and COVID-19 pandemic restrictions, the study had a high retention rate of 89% and an adherence rate of 83% [36,38]. These rates compare favorably with retention rates for other boxing-like exercise interventions [24]. For example, one study utilizing boxing exercises recruited 12/19 participants over 9-weeks with an adherence rate of 79% (+/- 15%) [39], while another study utilizing boxercise in an outpatient mental health setting reported a 50% retention rate [40] Notably, a virtual reality exergaming boxing study had a retention rate of 48% which may speak to the importance of an in-person group dynamic [41].

Reasons for absences for intervention sessions included holidays, inclement weather, transportation, and respiratory infections. Participants completed 100% of scales at each timepoint further highlighting the feasibility of this study. This is contrast to the lower completion rates within other non-contact boxing studies [40].

The high scores on the CSQ and User Experience scales, along with qualitative feedback, indicate a strong acceptability for the MBBT intervention. One major contributing factor to this positive reception is the unique nature of the program compared with traditional exercise interventions. To our knowledge, MBBT is the only non-contact boxing group intervention that incorporates principles of group therapy to build group cohesion [42]. Feedback highlighted the importance of safety, group cohesion, and leadership, indicating the value of the integrated therapeutic principles. Lastly, the program uses various elements to enhance exercise experience such as choice, music, instructor-led, group, and aromas. The results may indicate that patients are interested in exercise/behavioural interventions that are novel in helping with their mental health problems.

MBBT yielded significant reductions in depression, anxiety, and distress levels among participants, accompanied by overall global improvement. Empowering participants and promoting self-agency through behavioral approaches can lead to improved mental health outcomes by fostering a sense of control and self-efficacy, aligning with the secondary aim of our study [9]. The qualitative findings support the quantitative results. These preliminary quantitative findings, combined with qualitative themes emphasizing a cathartic release, increased self-esteem and self-agency, and the importance of community and leadership, are consistent with previous reports on the potential mental health benefits of non-contact boxing exercises [24]. This alignment suggests that the therapeutic elements of MBBT are likely integral to the observed improvements in mood, anxiety, and mindfulness. MBBT is distinct from other boxing programs since it is manualized, and incorporates aspects of mindfulness. The 79% increase in mindfulness in the current sample, and the reported themes of empowerment of emotions suggesting a unique role for mindfulness within MBBT. Further studies could explore if MBBT offers distinct advantages over conventional boxing or mindfulness programs such as MBSR.

Previous research has indicated the potential benefits of combining meditation and aerobic exercise (MAP training) for depression with studies reporting a potential increase in cognitive control processing and decreasing ruminative thought pattern [43]. The benefits of adjunctive physical activity with psychotherapy for post-traumatic stress disorder has also been previously reported [44]. Our study adds to this literature by specifically examining the effects of mindfulness-based principles embedded in non-contact boxing training on mental health outcomes.

### Limitations

The current study, while providing important feasibility and acceptability data, has major limitations. A significant limitation is the intervention being exclusively delivered by its developer (JB). With credentials as a trained psychiatrist, group therapy facilitator, MBSR certification holder and a Certified National Boxing Coach with 20-years of boxing experience, the instructor in the present study had a unique blend of expertise. This poses questions on the replicability of the study when future MBBT instructors, with varying levels of expertise, deliver the intervention. There is an inherent risk of biases such as investigator, expectation, selection, and publication biases when an intervention is delivered by its developer. Limited resources and the novelty of the intervention necessitated this direct involvement.

Pharmacotherapy may have influenced the results, and future studies should control for medication use or include it as a covariate to better isolate the effects of the MBBT intervention. Without a Treatment As Usual (TAU) or active control group, attributing observed changes solely to the MBBT intervention is challenging, as there is no baseline for comparison to rule out placebo effects, spontaneous remission, or other external factors. The inclusion of a control group, would provide a more robust comparison, allowing for a clearer interpretation of the intervention’s effectiveness and helping to isolate the specific effects of MBBT. In addition, including a third timepoint post-intervention would provide valuable information on the long-term effectiveness and durability of the MBBT intervention. Given that the primary focus of this study was feasibility and participant acceptability, the decision was made not to include a control group or third timepoint. Additionally, COVID-19 restrictions further limited our ability to implement these measures. We recognize that these are limitations affecting the clinical outcome of MBBT, and future studies should consider these additions to enhance the internal validity, generalizability, and robustness of the findings.

Additionally, our sample size was small which limits the generalizability of our findings. The MBBT intervention, held twice weekly with active exercise of around 45-60 minutes over 10 weeks, aligns well with CANMAT guidelines of exercise as an intervention for the treatment of MDD [6] However, when measured against more extensive guidelines, like those of the World Health Organization (WHO) that recommend a minimum of 150-300 minutes of moderate intensity aerobic activity per week, the MBBT active exercise time might appear on the lower end [45]. This discrepancy merits further exploration, especially in assessing if longer, or more frequent, sessions could offer amplified benefits.

Lastly, limited funding necessitated reliance on volunteer time, donated equipment, and conducting the study at a single site. Specifically, we utilized in-hospital resources such as the gymnasium, relied on in-kind support for research staff and study therapist. These constraints may have impacted the study’s replicability. Future studies should secure comprehensive funding to cover all aspects of the trial, including staffing, equipment, and multiple study sites to ensure generalizability and reduce potential researcher and selection bias.

## Conclusion

Recognizing the limitations of this study, our findings support the overall feasibility and acceptability of MBBT as an exercise/behavioural intervention in the outpatient setting for adults with depression or anxiety. These initial results support the potential for MBBT to be an accessible and acceptable approach to improve mental health outcomes. An adequately powered RCT with an appropriate control group is needed to evaluate the clinical benefits of MBBT. Future studies should also investigate the barriers to training non-specialist facilitators and implementing MBBT in different populations and settings.

## Supporting Information

S1 File Supportive Information. (DOC)

MBBT_TREND_Checklist (PDF)
