# Supplementary material for: Mindfulness-based (non-contact) boxing therapy (MBBT) for depression and anxiety: A feasibility study
Source: PLoS One. 2025 Feb 6;20(2):e0318364. doi: 10.1371/journal.pone.0318364 (PMC11801631; doi:10.1371/journal.pone.0318364)
Supplement: S1 File — (DOC) [file pone.0318364.s001.doc]

S1 File: Supportive Information:


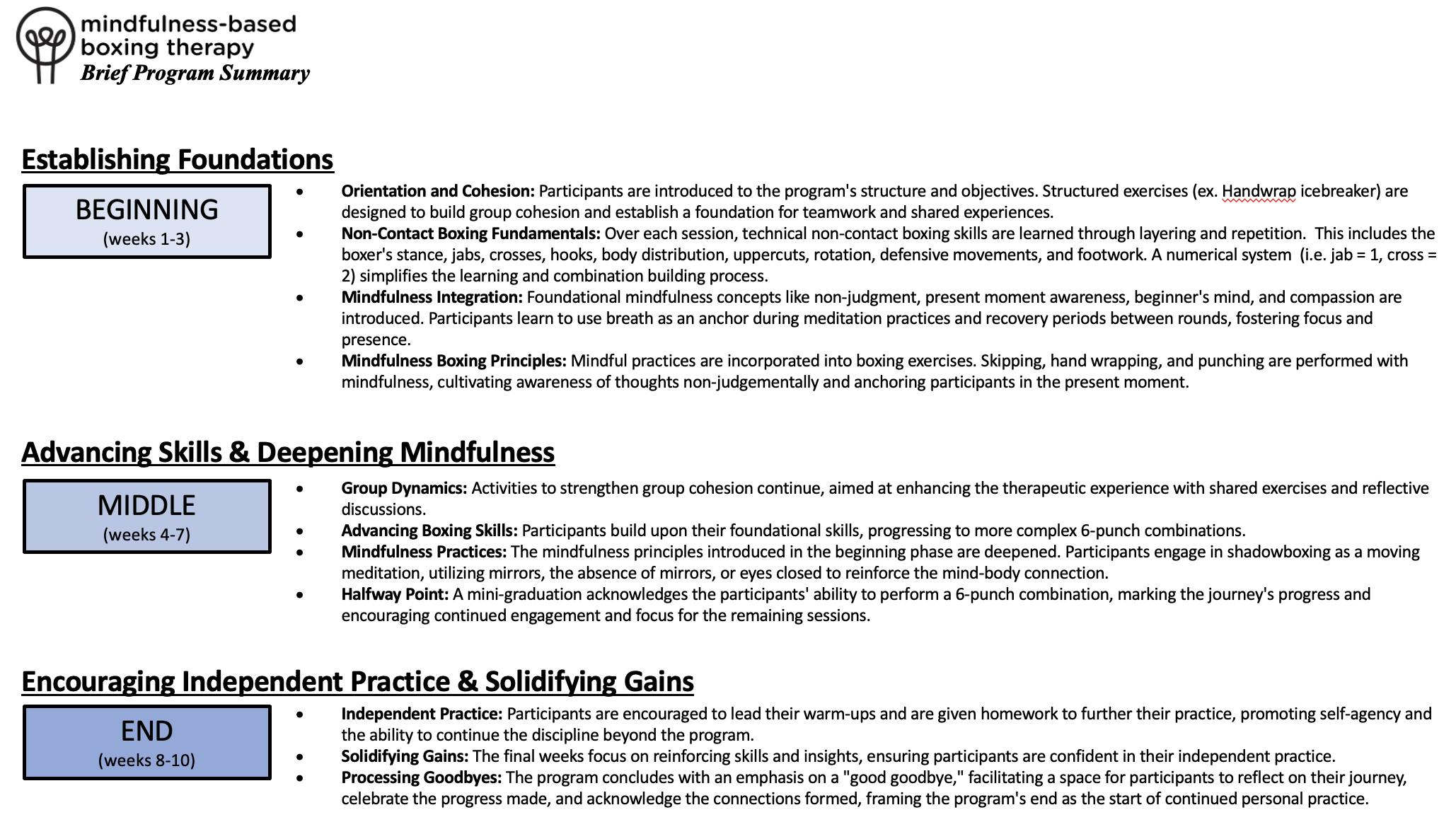


**S1 Fig. 3:** Summary of the phases within Mindfulness-Based (non-contact) Boxing Therapy

**Core Principles of Mindfulness-Based (non-contact) Boxing Therapy (MBBT)**

The MBBT program's unique approach lies in its holistic integration of non-contact boxing exercises, mindfulness, and group therapy principles, creating a novel pathway for participants to safely explore self-regulation and personal growth through physical activity and meditative practices. MBBT's scaffolding skills development utilizes strategic repetition to build a foundation that nurtures continuous growth and mastery. The facilitator carefully uses directed cues to enhance mindfulness and positive affirmations to motivate physical exertion, gently guiding participants beyond their perceived limitations in a secure environment. By beginning and ending with the ritual of mindful hand-wrapping and meditation, MBBT ensures that participants engage with, and then disengage from, their aggressive or anxious energies in a controlled, reflective manner. This sequence creates a space for both a cathartic release through exercise and a calming re-centering.

Further information on MBBT:

**1) Meditation**

Meditation prior to therapy or exercise can significantly enhance its benefits. In MBBT, meditation is used at the beginning and end of sessions to help participants center themselves, engage more fully with the physical exercises, and reflect on their experiences. Meditation practices in MBBT include handscan, mindful hand-wrapping, visualization, body scans, and counting breaths. These practices aim to ground participants in the present moment, reduce stress, and enhance their overall mental clarity and emotional stability.

**2) Activities to Strengthen Group Dynamics**

Drawing from the principles of group therapy as outlined by Yalom and Leszcz, MBBT emphasizes therapeutic factors such as instilling hope, fostering universality, and promoting group cohesion. As participants progress through increasingly challenging non-contact boxing exercises, a sense of cohesion and mutual support develops. Weekly check-ins allow participants to share experiences, fostering a supportive environment. Ideally, the shared journey through challenging exercises promotes a strong sense of community and belonging, which can significantly enhance participants' motivation and commitment to the program.

**3) Mindfulness Principles**

The integration of mindfulness into MBBT is based on the established benefits of mindfulness practices in enhancing emotional regulation, present-moment awareness, and non-judgmental acceptance. These principles are critical in reducing rumination, improving cognitive control, and fostering psychological resilience. By embedding mindfulness into the physical activity of non-contact boxing, we hypothesize that participants can achieve a synergistic effect where the mindfulness component enhances the mental health benefits of the physical exercise.

Within MBBT, a few examples of the principles of mindfulness incorporated include:

1. **Non-judgment:** Encouraging participants to observe their experiences without labeling them as good or bad, especially when learning new boxing combinations that may be challenging.
2. **Present-moment awareness:** Focusing on the current activity, whether it's the process of hand-wrapping, the feel of the gloves, or the sound of hitting the bag.
3. **Patience:** Encouraging participants to trust that their boxing skills will improve with consistent practice and time.
4. **Trust:** Helping participants develop confidence in their body’s ability to move at a pace that is right for them.
5. **Non-striving:** Emphasizing the importance of participating in the activity without the pressure to achieve or perform, allowing the experience of each MBBT session to unfold naturally.

The following outlines a few of the various mindfulness techniques incorporated into the MBBT framework:

1. **Handwrapping Ritual**: Each session begins and ends with a mindful handwrapping ritual. Participants are encouraged to bring their full awareness to the sensation of the wraps against their skin, using the act of wrapping as an opportunity to focus on the present moment. This practice helps participants engage with the session right from the start and serves as a grounding technique, preparing their minds and bodies for the physical exercise ahead. Mindful handwrapping emphasizes non-judgment, allowing participants to start their practice with a calm, centered mindset.
2. **Mindful Focus on Breath as an Anchor**: Throughout MBBT, the breath serves as an anchor to bring participants' attention back to the present moment between rounds of non-contact boxing exercises. As participants engage in high-intensity movements, they are encouraged to consciously regulate their breath, focusing on deep, controlled breathing to reset and reduce tension between exertions. In moments of rest, attention is directed toward the rise and fall of the breath, helping participants calm their nervous system and reconnect with their body. This repeated redirection of focus to the breath reinforces mindfulness and helps regulate emotions, particularly in moments of heightened physical activity.
3. **Awareness of Body and Stance**: During boxing exercises, participants are continuously reminded to bring their awareness to their body and stance. This includes paying attention to the alignment of their body, the positioning of their feet, and their overall posture. Cues from the facilitator, such as “keep your feet grounded” or “hands up” or “feel your balance” encourage participants to mindfully check in with their body during movements. This heightened bodily awareness fosters a deeper connection to their physical experience, promoting better form and safety while enhancing the overall mindfulness practice. The act of boxing becomes more than just physical exertion—it becomes a mental exercise in maintaining focused, non-judgmental awareness.
4. **Punching the Bag with Mindful Attention**: One of the key mindfulness practices integrated into MBBT is the focus on punching the bag with deliberate attention to sound, sensation, and intensity. Participants are instructed to vary their punch intensity, from light to strong, while maintaining focus on the sound each punch creates as it hits the bag. This attention to sound and impact helps participants stay engaged with the present moment. They are encouraged to notice the difference in sound, feel the physical sensation of their glove hitting the bag, and remain aware of the tension and release in their muscles. This practice promotes mindfulness by grounding participants in the sensory experience of boxing and reducing distractions or ruminative thoughts.
5. **Bringing Attention Back to the Present Moment**: Throughout the session, participants are regularly reminded to bring their attention back to the present moment if their minds wander. For example, if a participant becomes distracted or begins to think about the outcome of their performance, the facilitator may say, “Focus on your stance,” or “Notice your breath.” These gentle reminders help participants re-engage with the present moment and enhance their mindfulness skills. Boxing, with its repetitive and rhythmical movements, offers many opportunities to practice returning to the present.
